# The Early Diagnosis of Cancer of the Pylorus

**Published:** 1901-11-23

**Authors:** 


### THE EARLY DIAGNOSIS OF CANCER OF THE PYLORUS.

The results of operations for the removal of cancer of the stomach have been so poor that very few surgeons are in the habit of recommending their performance, at least in the circumstances in which this disease is usually diagnosed, *i.e.* at a more or less advanced stage. Nevertheless the results have not been entirely disheartening, and the very serious question arises whether they might not even become encouraging if it were possible to operate at a far earlier period than has hitherto been practicable. This brings up the further question whether, if one were to investigate suspicious cases by aid of an exploratory operation, one might not be able to discover the disease at such a stage that it might be capable of removal—using the word "removal" in the same meaning as that in which it is used in reference to cancer of, say, the breast. Before we can answer this we must be able to form some sort of idea as to the kind of operation which such a complete removal of a cancer of the pylorus would involve. The remarks made by Mr. Moynihan at the last meeting of the Clinical Society indicate pretty clearly the direction in which surgery must advance, if removal of the pylorous is to become a useful operation, and tend strongly to suggest that if the same principles can be applied in the removal of cancer of the pylorus as have proved so advantageous in dealing with cancer of the breast, a new era will be opened in the treatment of this class of diseases. A new importance is thus given to any measure by which a very early diagnosis may be arrived at. He showed, by an examination of the records, that where recurrence took place after operation for the removal of malignant disease of the stomach, the return of the growth was almost always local; as, indeed, was to be expected, seeing the stage of the disease at which operations have been performed, and the impossibility of removing the whole of the infected area. He also pointed out that for an operation to be successful in preventing recurrence, it must be based upon a knowledge (1) of the mode of invasion of the stomach by the primary growth; (2) of the lymphatic distribution in the stomach; and (3) of the position of the glands into which the vessels drain; and the major part of his paper was devoted to a careful description of these details. The outcome of his remarks was that in order to ensure complete removal of the primary growth, of the infiltrated lymphatic vessels, and of the glands into which those vessels drain, it would be necessary to remove the stomach as far up on the lesser curvature as the point of abutment of the coronary artery, and on the greater curvature as far as the gastro-splenic omentum, and to remove the first part of the duodenum. After removal of the portion of the stomach included within these limits, the anastomosis between the small intestine and the remaining portion of the stomach (an area at the cardiac end comprising the greater tuberosity of the stomach, which he speaks of as the "isolated area" in consideration of its lymphatic connections) might be made by end-to-end approximation, or by suture of the cut ends and the performance of a separate gastro enterostomy. The importance of Mr. Moynihan's paper is that it puts plainly before us the sort of operation (its extent and its direction) which must be undertaken before removal of cancer of the pylorus can claim to stand upon the same footing as modern operations for cancer of the breast—and this even in cases where operation is performed at a far earlier stage than has usually been possible up to the present time. If such an operation as Mr. Moynihan describes is necessary in an early case—and it must be necessary if even a small cancer of the breast demands the clearing of the axilla—we can well understand the poor success which has so far followed operation in those more or less advanced cases which have mostly come into the surgeon's hands. In regard to diagnosis what Mr. Moynihan says is that in the case of a patient under treatment for gastric trouble at an age when one might expect to meet with cancer of the stomach, if there were found absence of free hydrochloric acid in the stomach, then, whatever the physical signs and clinical condition might be, it was his practice to advise an exploratory incision.

